# Platelet-Rich Plasma in Cardiovascular Regeneration: Mechanistic Insights, Technological Innovations, and Future Directions

**DOI:** 10.31083/RCM39383

**Published:** 2025-07-28

**Authors:** Ju Tian, Jing Chen, Xiuling Lai, Jing Ding, Jie Sun, Dandan Shi, Xiaoying He, Xingqi Chen

**Affiliations:** ^1^Department of Plastic Surgery, ZhongshanCity People’s Hospital, 528400 Zhongshan, Guangdong, China; ^2^Department of Surgical Anesthesia, ZhongshanCity People’s Hospital, 528400 Zhongshan, Guangdong, China; ^3^Department of Cardiovascular, ZhongshanCity People’s Hospital, 528400 Zhongshan, Guangdong, China

**Keywords:** platelet-rich plasma (PRP), cardiovascular regenerative medicine, angiogenesis, myocardial repair, nanotechnology-driven delivery

## Abstract

Platelet-rich plasma (PRP), an autologous concentrate of platelets and bioactive molecules, has emerged as a promising regenerative therapy in cardiovascular medicine. The potential of PRP extends beyond hemostasis to include myocardial repair, angiogenesis, and immunomodulation. This review explores the biological mechanisms of PRP, its clinical applications in ischemic heart disease, peripheral artery disease, and inflammatory cardiopathies, and addresses challenges in standardization and translation. PRP exerts therapeutic effects through three primary mechanisms: promoting angiogenesis by stimulating endothelial cell proliferation and migration, exerting anti-inflammatory and immunomodulatory effects by balancing cytokine release, and enhancing myocardial repair and functional recovery by activating resident cardiac progenitor cells. Despite the promise of PRP, challenges such as variability in PRP composition due to differences in preparation methods and safety concerns remain. To overcome these barriers, precision engineering and cross-disciplinary integration are crucial. Innovations such as nanotechnology-driven targeted delivery systems and clustered regularly interspaced short palindromic repeats (CRISPR)-edited exosomes offer mechanism-specific interventions. Artificial intelligence (AI)-driven approaches utilizing single-cell RNA sequencing data can enable personalized treatment strategies, while closed-loop systems minimize batch-to-batch variability. Collaborative efforts between clinicians, engineers, and regulators are essential to establish global standards for exosome characterization. PRP-based therapies hold immense promise for revolutionizing cardiovascular regenerative medicine by modulating angiogenesis, inflammation, and myocardial repair. By embracing these advanced technologies and interdisciplinary approaches, PRP can transition from an empirical treatment to a data-driven, mechanism-specific intervention, ultimately redefining the future of cardiovascular care.

## ​1. Introduction​

Cardiovascular diseases (CVDs), encompassing myocardial infarction (MI), peripheral artery disease (PAD), and atherosclerosis, rank among the leading causes of mortality globally, with coronary artery disease (CAD) being particularly notable for its potential to precipitate MI [1]. Following MI, the heart’s intrinsic regenerative capacity is constrained, resulting in irreversible loss of cardiomyocytes and persistent impairment of cardiac function. This has spurred intensive research in cardiovascular medicine to identify therapeutic strategies capable of stimulating cardiac cell renewal and restoring organ performance. Despite advancements in pharmacological interventions, revascularization procedures, and lifestyle modifications, current clinical approaches predominantly focus on mitigating risk factors and alleviating symptoms but inadequately address the fundamental pathophysiological processes driving post-infarction remodeling. These processes include myocardial fibrosis, endothelial dysfunction, and persistent inflammatory responses. This critical therapeutic gap highlights the urgent need for innovative regenerative strategies capable of restoring tissue homeostasis and promoting authentic functional recovery in the damaged myocardium.

In recent years, platelet-rich plasma (PRP), an autologous blood product abundant in growth factors, has garnered significant attention due to its hemostatic properties and tissue reparative potential [2]. PRP contains a constellation of bioactive molecules and chemotactic factors that are released upon platelet activation, demonstrating robust tissue regenerative capacities. Notable advancements have been achieved in applying PRP across diverse regenerative medicine domains, with particularly promising prospects in cardiovascular therapeutics [3, 4]. By delivering critical cytokines and growth factors such as vascular endothelial growth factor (VEGF) and platelet-derived growth factor (PDGF), PRP facilitates cardiomyocyte regeneration and angiogenesis. Emerging evidence further suggests that PRP may enhance myocardial repair through novel mechanisms like Piezo-type mechanosensitive ion channel component 1 (Piezo1) channel activation [4], representing a multifaceted approach to cardiac tissue restoration.

However, challenges persist regarding PRP preparation standardization, therapeutic variability, and potential adverse effects. To fully harness the regenerative potential of PRP in cardiac cell therapy, there is a critical need for integrated guidelines, continuous research, and enhanced clinical translation. This comprehensive review aims to elucidate recent advancements in PRP research within cardiovascular medicine, particularly focusing on its applications in cardiac cell regeneration and precision therapeutics, while addressing existing hurdles and future research trajectories.

## 2. Mechanisms of Action of PRP in Cardiovascular Disease Therapeutics

The therapeutic efficacy of PRP stems from its unique composition, including platelets, fibrin, and leukocytes. Upon activation, platelets release a repertoire of bioactive molecules such as PDGF, ​transforming growth factor-β (TGF-β), and VEGF, which serve as critical regulators of cellular proliferation, differentiation, and extracellular matrix remodeling [5]. In the context of cardiovascular diseases, PRP exerts multifaceted biological effects through three primary mechanisms (Table 1).

**Table 1.** **Mechanisms of action of PRP in cardiovascular regeneration​**.

Mechanism category	Key molecular mediators & signaling pathways	Functional impact
1. Angiogenesis	- VEGF: Direct endothelial proliferation/migration, VEGFA/VEGFR2 activation, MMP2/9 upregulation (ischemia)	Stimulates microcirculatory remodeling, enhances blood perfusion in ischemic myocardium/peripheral tissues
	- PDGF: Integrin-AKT signaling for cell adhesion/migration, PDGFRβ-regulated pericyte stabilization	Stabilizes nascent vessels, prevents leakage/fibrosis, supports arteriogenesis
	- TGF-β: ECM remodeling via SMAD2/3 pathway, balanced collagen deposition	Limits pathological fibrosis, preserves ventricular compliance
2. Anti-Inflammation	- Anti-inflammatory cytokines: IL-10, TGF-β suppress TNF-α/IL-6	Biphasic modulation:
		∙ Acute phase: Inhibits M1 macrophage polarization
		∙ Chronic phase: Promotes M2 repair phenotype
	- Leukocyte modulation: LR-PRP (high leukocyte) enhances IL-1Ra/IL-4 secretion	Tailored formulations for phase-specific inflammation (e.g., LR-PRP for acute MI, LP-PRP for chronic CAD)
3. Myocardial Repair	- Growth factor synergy: VEGF-C (coronary endothelial cloning), PDGF-BB (PDGFRβ signaling in fibroblasts enhances collagen deposition and pericyte stabilization)	Activates resident cardiac progenitor cells (CPCs), improves electrical coupling, reduces scar formation
	- Mechanotransduction: Platelet microparticles (PMVs) transmit biomechanical signals via Piezo1 channels	Induces cardiomyocyte dedifferentiation/mitochondrial biogenesis

CAD, coronary artery disease; ECM, extracellular matrix; IL-6, interleukin-6; IL-10, interleukin-10; LR-PRP, leukocyte-rich PRP; LP-PRP, leukocyte-poor PRP; MI, myocardial infarction; PDGF, platelet-derived growth factor; PRP, platelet-rich plasma; TGF-β, transforming growth factor-β; TNF-α, tumor necrosis factor-α; VEGF, vascular endothelial growth factor; VEGFA, vascular endothelial growth factor A; VEGFR2, vascular endothelial growth factor receptor 2; MMPs, matrix metalloproteinases; AKT, Protein Kinase B; PDGFRβ, platelet-derived growth factor receptor beta; SMAD, Sma- and Mad-related proteins.

### 2.1 Promoting Angiogenesis

The synergistic effects of VEGF and PDGF in PRP play pivotal roles in stimulating endothelial cell proliferation and migration, with VEGF directly promoting these processes and facilitating microcirculation reconstruction through the activation of the vascular endothelial growth factor A (VEGFA)/vascular endothelial growth factor receptor 2 (VEGFR2) pathway, as evidenced in models of diabetic foot ulcers [6], while also enhancing capillary formation and blood flow restoration in ischemic tissues by upregulating MMP2/9 and Estrogen Receptor Alpha (ERα)/Rho-associated, coiled-coil containing protein kinase 2 (ROCK2) signaling pathways [7]. PDGF, on the other hand, promotes cell migration and adhesion through Protein Kinase B (AKT) activation within the integrin signaling pathway, exhibiting superior effects compared to PDGF-BB alone, and its receptor, platelet-derived growth factor receptor beta (PDGFRβ), regulates pericyte survival and migration, thereby stabilizing newly formed blood vessels [8]. Additionally, TGF-β, stored in a latent form within the extracellular matrix (ECM), modulates ECM deposition and fibrosis repair upon activation through the TGF-β1/SMAD2/3 pathway [9]. In the context of MI, VEGF-C enhances post-MI angiogenesis by promoting clonal expansion of coronary endothelial cells, while PDGF-BB and TGF-β1 contribute to the stabilization of the neovascular network by regulating ECM remodeling and pericyte coverage [10]. Collectively, these growth factors drive neovascularization, improving blood perfusion to ischemic tissues, particularly beneficial for patients with peripheral artery disease or myocardial infarction [11].

### 2.2 Anti-Inflammatory and Immunomodulatory Effects

PRP demonstrates remarkable anti-inflammatory and immunomodulatory capabilities rooted in its complex cytokine and growth factor composition [12, 13, 14]. By releasing anti-inflammatory mediators like interleukin-10 (IL-10) and TGF-β while suppressing pro-inflammatory cytokines such as tumor necrosis factor-α (TNF-α) and interleukin-6 (IL-6), PRP effectively balances inflammatory responses. Additionally, its growth factors including VEGF, PDGF, and TGF-β—not only drive tissue repair but also indirectly reduce inflammation by accelerating healing processes. This dual action is further enhanced through reactive oxygen species scavenging and modulation of immune cell phenotypes, such as shifting macrophages from M1 to M2 and regulating T-cell responses to prevent excessive immune activation [15]. In inflammatory diseases such as osteoarthritis, PRP demonstrates therapeutic effects through its dual actions, not only alleviating pain (associated with reduced levels of TNF-α and IL-6) [16, 17, 18] but also promoting tissue repair. A study has further revealed that leukocyte-rich PRP (LR-PRP) exhibits stronger anti-inflammatory properties compared to leukocyte-poor PRP (LP-PRP), as evidenced by higher levels of IL-1 and IL-4 [19]. Collectively, these findings suggest that PRP restores inflammatory balance through a “bidirectional regulatory” mechanism—simultaneously enhancing anti-inflammatory effects and suppressing pro-inflammatory responses [20]. This characteristic endows PRP with potential applications in the treatment of various inflammation-related diseases [21].

PRP’s therapeutic potential shines in cardiovascular pathologies where inflammation and immune dysregulation are pivotal. In conditions like atherosclerosis, myocardial infarction, and peripheral artery disease, PRP limits plaque inflammation, reduces infarct size, and promotes neovascularization in ischemic tissues [22]. However, its efficacy hinges on formulation nuances—such as leukocyte content—and delivery methods, which influence cytokine bioavailability and immune cell trafficking.

### 2.3 Myocardial Repair and Functional Recovery

By activating resident cardiac progenitor cells and enhancing cardiomyocyte survival, PRP promotes myofiber alignment and improves contractile function in infarcted hearts. Preclinical studies demonstrate that PRP therapy increases left ventricular ejection fraction (LVEF) and holds promise for structural and functional restoration of damaged myocardium [23, 24].

Research indicates that PRP may enhance myocardial repair through novel mechanisms, such as Piezo1 channel activation [4]. Piezo1, a mechanically sensitive cation channel, facilitates an increase in intracellular Ca^2+^ levels [25]. PRP activates the AKT pathway within the integrin signaling cascade via PDGF receptor tyrosine kinase, thereby promoting the adhesion and migration of cardiac progenitor cells (CPCs) [26]. Concurrently, the mechanically sensitive ion channel Piezo1, upon sensing mechanical stimuli in CPCs, mediates calcium influx, enhancing their proliferative and differentiative capabilities through the Ca^2+^/Hypoxia-inducible factor 1α (HIF-1α)/VEGF signaling axis [27], and modulates paracrine functions to facilitate myocardial repair [28]. In endothelial cells (ECs), the synergistic effects of VEGF and PDGF released by PRP are notable: VEGF activates the endothelial nitric oxide synthase (eNOS)/nitric oxide (NO) and PI3K/AKT pathways by binding to VEGFR2 [29, 30], while PDGF enhances cell migration through an integrin-dependent mechanism. Together, they regulate Piezo1-mediated Ca^2+^ influx, dynamically balancing angiogenesis (via the HIF-1α/VEGF and FGF2 pathways) [31] and endothelial permeability (modulated by VEGF-induced barrier function) [32]. In pathological microenvironments, hypoxia exacerbates VEGF secretion by upregulating Piezo1 and HIF-1α expression, whereas inflammation disrupts EC function through the ERK/p38/STAT1 signaling pathway [33]. However, the activation of the Piezo-type mechanosensitive ion channel component 1 - Calcium/calmodulin-dependent protein kinase II - Focal adhesion kinase/Proto-oncogene tyrosine-protein kinase Src - Yes-associated protein (Piezo1-CaMKII-FAK/Src-YAP) axis alleviates endothelial inflammation in atherosclerosis [34], underscoring PRP’s dual cell-specific mechanisms—promoting regeneration in CPCs via the PDGF-integrin-Piezo1 axis and coordinating vascular homeostasis in ECs [35].

## 3. Specific Applications of PRP in Cardiovascular Disease Therapy 

### 3.1 The Application of PRP in Ischemic Heart Disease

PRP has emerged as a promising therapeutic option for ischemic heart disease, including conditions like angina and MI [36, 37]. By delivering a concentrated cocktail of growth factors such as PDGF-BB, TGF-β, and VEGF directly to ischemic myocardium, PRP activates endogenous repair mechanisms. This includes stimulation of cardiomyocyte proliferation, angiogenesis, and modulation of fibroblast activity to minimize scar formation [4, 36, 37, 38, 39].

In severe angina patients, PRP has shown good safety profiles while promoting myocardial repair through accelerated angiogenesis and mitigation of oxidative stress. The presence of anti-inflammatory cytokines in PRP also aids in reducing inflammation and apoptosis, further protecting cardiac tissue from ischemic injury. Additionally, PRP’s ability to reduce E-selectin expression improves endothelial function, which is crucial for cardiovascular health [40]. An experimental study has highlighted that PRP derived from young donors can rejuvenate aged bone marrow mesenchymal stem cells (BMSCs), enhancing their therapeutic efficacy in ischemic heart disease [41]. These findings underscore PRP’s potential as a dynamic therapeutic tool in addressing ischemic heart disease and its complications.

### 3.2 Vascular Regeneration and Repair

Emerging therapeutic strategies leveraging bioactive agents and bioengineered constructs have demonstrated promising outcomes in enhancing vascular regeneration and myocardial repair. A notable synergistic approach combines transmyocardial revascularization (TMR) with PRP to augment cardiac function post-coronary artery bypass grafting (CABG). This combinatorial therapy capitalizes on TMR’s mechanical creation of channels in ischemic myocardium and PRP’s pro-angiogenic growth factors, collectively improving myocardial perfusion and contractile function [42]. PRP has emerged as a versatile therapeutic with multi-faceted benefits. Clinical trials in patients with severe angina highlight its remarkable safety profile, while preclinical studies underscore its regenerative potential. For instance, PRP injection in MI patients significantly improves cardiac function and quality of life by stimulating neovascularization and inhibiting scar formation [43]. Additionally, PRP accelerates cardiac healing by mobilizing reparative cells and modulating inflammation [3].

In PAD, where arterial occlusion heightens amputation risks, bioengineered scaffolds incorporating mast cells (MCs) and PRP within chitosan matrices have demonstrated therapeutic angiogenesis. A rat hindlimb ischemia model revealed that MCs- and PRP-loaded scaffolds enhanced vascular density and restored blood flow, offering a promising strategy for ischemic tissue revascularization [44].

Collectively, these studies emphasize the translational potential of combinatorial therapies and bioengineered constructs in addressing cardiovascular pathologies. By harnessing the angiogenic and immunomodulatory properties of PRP, alongside stem cell mobilization strategies, clinicians may achieve superior outcomes in myocardial repair and peripheral vascular regeneration.

### 3.3 Anti-Inflammatory and Immunomodulatory Effects

Beyond its regenerative and angiogenic properties, PRP demonstrates immunomodulatory capacities that mitigate post-injury inflammation. Leukocytes and growth factors within PRP coordinate an anti-inflammatory response to limit tissue damage. In inflammatory cardiovascular pathologies such as myocarditis and pericarditis, PRP suppresses pro-inflammatory cytokine release, alleviates clinical symptoms, and promotes tissue repair. Notably, PRP-derived leukocytes secrete anti-inflammatory cytokines like IL-10 while paracrine signaling inhibits pro-inflammatory mediators such as TNF-α and IL-6. This immunoregulatory mechanism provides distinct therapeutic benefits in inflammatory heart diseases. Preclinical studies of autoimmune myocarditis show PRP therapy reduces myocardial macrophage infiltration by 50% and decreases fibrotic regions by 35% via TGF-β/Smad signaling pathway downregulation [3]. Additionally, PRP’s immunomodulatory actions enhance transplanted stem cell survival and engraftment efficiency, fostering a synergistic environment for tissue restoration.

## 4. Current Issues and Challenges

Despite significant advancements in PRP formulation and delivery systems, several critical issues persist in translating its regenerative potential into clinical practice.

### 4.1 Efficacy of PRP Treatment and Influencing Factors 

PRP exhibits therapeutic potential in cardiovascular regeneration by releasing bioactive molecules such as VEGF, PDGF, and TGF-β, which promote angiogenesis, inhibit inflammation, and enhance myocardial repair. Preclinical studies confirm its ability to improve blood perfusion in ischemic myocardium and accelerate postoperative wound healing [45, 46].

#### 4.1.1 Standardization of Production 

The standardization of PRP production is crucial for ensuring consistent clinical outcomes, yet significant variability persists across preparation techniques, centrifugation parameters, activation methods, and quality control standards. To address this, consensus guidelines defining optimal protocols and quality control metrics, along with standardized preparation kits and automated devices, are needed to reduce operator-dependent variability. Despite the existence of general recommendations provided by the International Society on Thrombosis and Haemostasis (ISTH) [47], there remains a notable absence of universally accepted standards for optimizing platelet activation and managing leukocyte content. This lack of standardization not only undermines therapeutic outcomes but also complicates the interpretation of clinical trial results.

The therapeutic efficacy of PRP is profoundly influenced by its preparation methodology, which encompasses various factors such as centrifugation protocols, platelet concentration levels, activation methods, and the inclusion or exclusion of leukocytes [48, 49, 50]. For example, Single-spin protocols (lower g-forces, shorter duration) may preserve higher levels of TGF-β1 (pro-fibrotic) due to reduced platelet lysis, potentially exacerbating myocardial scarring in chronic ischemia [51]. Double-spin protocols (higher g-forces, prolonged centrifugation) enrich platelet concentration but risk degrading labile TGF-β3 (anti-fibrotic) and over-concentrating TGF-β2 (context-dependent effects), complicating outcomes in myocarditis or wound healing [52].

#### 4.1.2 Immune Microenvironment Adaptation 

Selection of PRP subtypes (e.g., LR-PRP for acute inflammation, monocyte-rich PRP for M2 macrophage polarization) must align with disease stages. The inclusion of leukocytes in PRP formulations presents a dual-edged therapeutic impact. LR-PRP may amplify antimicrobial defense via reactive oxygen species and neutrophil extracellular trap (NET) formation, yet paradoxically exacerbate tissue injury in inflammatory contexts through these same mechanisms. Contrary to expectations, clinical evidence suggests that LR-PRP exhibits elevated anti-inflammatory mediator expression compared to LP-PRP, potentially benefiting patients with chronic low-grade inflammation, such as those with long-standing knee osteoarthritis [19]. Monocyte-rich PRP, a subtype of leukocyte-rich PRP characterized by high CD14+CD16+ monocyte concentrations, demonstrates superior immunomodulatory potential compared to LP-PRP. However, its pro-inflammatory properties may paradoxically aggravate pathologies involving dysregulated immune activation [53, 54]. These findings underscore the necessity for disease-specific PRP optimization: In myocarditis, LR-PRP’s NETosis-inducing capacity might worsen inflammatory cascades. In chronic wounds, monocyte-rich PRP’s M2 macrophage-polarizing effects could enhance tissue repair.

Tailoring PRP leukocyte composition to the host immune microenvironment is critical for maximizing therapeutic benefits while minimizing adverse effects.

#### 4.1.3 Patient Heterogeneity

Age, comorbidities (e.g., diabetes), and genetic backgrounds alter platelet activity and growth factor receptor expression, contributing to variable therapeutic responses.

In summary, the clinical outcomes of PRP are affected by numerous complex factors, including patient characteristics (age, comorbidities, genetics), disease type and stage (acute/chronic, fibrosis level), PRP’s biological features (growth factor ratios, platelet activation, cellular makeup), administration methods (route, dose, frequency), combined treatments (biomaterial compatibility, cellular enhancement, physical interventions), immune microenvironment dynamics (macrophage polarization, immunosuppression), storage conditions (cryopreservation, anticoagulant choice), and efficacy assessment limitations (surrogate endpoint bias, heterogeneity) [55, 56, 57]. The variability in PRP’s efficacy arises from the dynamic interaction of these multidimensional factors (e.g., patient traits → immune environment → administration techniques). To improve PRP therapy, future efforts should focus on standardizing preparation (e.g., uniform platelet count thresholds), precise patient selection (e.g., biomarker screening), and intelligent delivery systems (e.g., controlled-release scaffolds).

### 4.2 Potential Side Effects of PRP

The side effects of PRP are generally mild and transient. However, it is crucial to remain vigilant about potential risks, which include: temporary redness, swelling, pain, or bruising at the local injection site; pathological reactions caused by an excess of growth factors, such as neointimal hyperplasia due to excessive VEGF stimulation leading to vascularproliferation (especially increasing the risk of restenosis in peripheral arterial disease), or PAD [58], or myocardial/tissue fibrosis induced by an overabundance of TGF-β [59]; in extremely rare cases, it may activate systemic pro-inflammatory mediators (e.g., IL-1β) or trigger immune imbalance (e.g., high-leukocyte PRP activating M1 macrophages). Additionally, there is a risk of thrombosis due to the high activity of platelets, particularly in high-risk populations such as those with atrial fibrillation. Therefore, in clinical practice, it is essential to strictly adhere to the principles of individualized assessment and dynamic monitoring.

### ​4.3 Targeted Delivery and Mechanism Specificity​

Current PRP formulations lack precise spatiotemporal control over bioactive molecule release, which is critical given the context-dependent actions of these factors. For example, while VEGF in PRP promotes angiogenesis in ischemic myocardium, its uncontrolled delivery risks disrupting vascular homeostasis. Similarly, the immunomodulatory effects of leukocytes in PRP are context-specific, aiding recovery in acute injury but potentially exacerbating chronic fibrosis. Furthermore, recent findings highlight the detrimental impact of persistently elevated growth differentiation factor 11 (GDF11) levels during aging [60], which may contribute to the loss of cardioprotective mechanisms and poor outcomes in elderly patients following acute myocardial infarction. These insights underscore the need for mechanism-specific biomarkers and targeted delivery strategies to optimize PRP’s therapeutic efficacy across diverse clinical scenarios.

## ​5. Future Direction

To fully harness the therapeutic potential of PRP in cardiovascular medicine, a transformative shift towards precision regenerative therapies is imperative. This involves integrating advanced technologies and cross-disciplinary approaches to address current limitations in PRP formulation and delivery.

### 5.1 Operational Standardization, Species-Specific Considerations, and Long-Term Safety in PRP Therapy for Cardiovascular Regeneration​

While PRP’s therapeutic potential is evident, its clinical translation is hindered by methodological heterogeneity in preparation protocols. To address this, we propose adopting Food and Drug Administration (FDA)-like regulatory frameworks for PRP manufacturing, including: Define minimal platelet concentrations (e.g., ≥1 × 10^9^ platelets/µL) based on clinical trial data (e.g., ISTH guidelines) to ensure therapeutic efficacy. Stratify PRP formulations into LR-PRP vs. LP-PRP​ based on disease context (e.g., LR-PRP for acute inflammation, LP-PRP for chronic ischemia) to balance pro-/anti-inflammatory effects. Standardize agonists (calcium chloride vs. thrombin) and activation times to control growth factor release kinetics (e.g., sustained release via collagen scaffolds). These measures would align PRP production with Good Manufacturing Practice principles, enhancing inter-study comparability and regulatory approval pathways.

Preclinical studies often overlook fundamental differences between rodent and human cardiac biology. For example: Unlike rodents, adult human cardiomyocytes exhibit minimal regenerative capacity, rendering rodent models of PRP-induced LVEF improvement poorly predictive of human outcome. Rodent hearts require microliter volumes of PRP, whereas clinical translation demands milliliter-scale delivery with maintained bioactivity. Future studies must prioritize large-animal models (e.g., porcine MI) to bridge this gap and validate PRP’s efficacy in systems recapitulating human cardiac physiology.

PRP’s therapeutic value remains undefined relative to established interventions: While PRP enhances TMR-CABG outcomes, its combination with stem cell-TMR has not been evaluated for synergistic effects on myocardial regeneration. Autologous PRP is inexpensive compared to exosome-based therapies, but its short half-life (<72 hours) may necessitate repeated injections, raising long-term costs. Health-economic analyses and head-to-head RCTs are urgently needed to define PRP’s niche in the regenerative therapeutics landscape.

The paucity of long-term data undermines confidence in PRP’s safety profile: Early studies report transient arrhythmias post-PRP injection, but ≥5-year risks of sustained arrhythmias or scar destabilization remain unexplored. While PRP reduces infarct size acutely, its impact on collagen cross-linking and diastolic dysfunction over decades is unknown. Registry studies tracking ≥10-year outcomes in PRP-treated cohorts are critical to validate durability and rule out delayed complications.

### 5.2 Predictive Biomarkers for Personalized Medicine

Current PRP protocols lack standardized stratification tools, such as baseline inflammatory biomarkers (e.g., high-sensitivity C-reactive protein, hs-CRP) and platelet function assays (e.g., VerifyNow® P2Y12), to identify responder populations. Elevated hs-CRP or TNF-α levels may predict adverse responses to leukocyte-rich PRP, necessitating leukocyte depletion in pro-inflammatory phenotypes. VerifyNow® P2Y12 testing can optimize platelet activation protocols by identifying patients with impaired platelet reactivity, while integrating multi-omics signatures (e.g., platelet RNA profiles) into clinical.

### 5.3 Nanotechnology-Driven Targeted Therapy

Nanotechnology is revolutionizing cardiovascular therapies by enhancing PRP efficacy through bioengineering. Nanoparticles serve as precision delivery vehicles for bioactive molecules in PRP, enabling targeted delivery to injured tissues. As detailed in Table 2, designing nanocarriers involves target identification, material selection, growth factor loading, and *in vivo* validation to optimize specificity, stability, and therapeutic outcomes. This approach minimizes off-target effects and maximizes regenerative potential.

**Table 2.** **Nanocarrier design for PRP delivery​**.

Step	Key actions	Purpose
1. Target identification	Use single-cell sequencing to identify endothelial/pericyte receptors (e.g., VEGFR2).	Optimize ligand-receptor binding specificity.
2. Material selection	Choose biocompatible carriers (e.g., lipid nanoparticles, Poly lactic-co-glycolic acid).	Balance stability, payload capacity, and degradation kinetics.
3. Growth factor loading	Encapsulate/immobilize PDGF, VEGF, TGF-β via physical adsorption or covalent bonds.	Protect labile factors; control release kinetics.
4. Target identification	Use single-cell sequencing to identify endothelial/pericyte receptors (e.g., VEGFR2).	Optimize ligand-receptor binding specificity.
5. *In vivo* validation	Test in ischemic heart disease models (e.g., mouse MI) for biodistribution and efficacy.	Validate targeting efficiency and therapeutic outcomes.

Emerging studies, exemplified by Vilella-Figuerola *et al*. [61], underscore the necessity of characterizing platelet-derived extracellular vesicles across diverse cardiac pathologies, revealing nanotechnology’s potential to tailor PRP therapies to distinct clinical scenarios. While advanced nanomaterials integrate stimuli-responsive mechanisms (e.g., pH-sensitive linkages, magnetic guidance) to enhance delivery precision, current proposals inadequately address off-target risks such as pulmonary accumulation or immune activation. Similarly, CRISPR-edited exosomes—repurposed as nanocarriers for gene-silencing therapies—raise concerns about unintended genomic effects, including off-target editing in bystander cells or genomic instability, which remain unvalidated in preclinical models. These limitations necessitate rigorous evaluation of nanocarrier biocompatibility, CRISPR fidelity, and scalable delivery systems to bridge translational gaps in cardiovascular PRP applications.

### 5.4 Single-Cell RNA Sequencing and AI-Driven Personalization

While single-cell RNA sequencing (scRNA-seq) and AI offer transformative insights into PRP cellular heterogeneity and platelet subset functional specialization in cardiovascular disease, current applications overlook critical clinical variables such as patient comorbidities (e.g., diabetes-induced platelet hyperreactivity) and circulating factors (e.g., GDF11 modulating stem cell activity). Integrating platelet subpopulations (e.g., CD41+CD62P+ subsets linked to thrombotic vs. reparative phenotypes) with clinical outcomes (e.g., post-MI neointimal hyperplasia or diastolic dysfunction) is essential to refine predictive models. Without accounting for these confounders, AI-driven PRP personalization risks misclassifying high-risk cohorts, such as diabetic patients with GDF11-driven platelet dysfunction, underscoring the need for multi-omic integration to anchor therapeutic decisions in biological and clinical reality.

### 5.5 Closed-Loop Production and Automated Quality Control

Standardizing PRP preparation is crucial for ensuring safety and efficacy. Microfluidic devices and closed-loop centrifugation systems (Table 3) minimize batch variability, ensuring consistent platelet counts and growth factor profiles. AI-powered sensors can monitor critical parameters, such as pH and temperature, in real-time during PRP processing. Automated quality control further guarantees that each batch meets therapeutic standards, reducing human error and variability.

**Table 3.** **Closed-loop production system​**.

Step	Key actions	Purpose
1. Blood collection	Draw autologous whole blood + anticoagulant (e.g., citrate).	Ensure sterility and prevent clotting.
2. Automated centrifugation	Dual-stage spin (low-speed Red Blood Cell removal + high-speed platelet enrichment).	Standardize platelet concentration and purity.
3. Real-time monitoring	Sensor-based Quality Control (QC): Platelet count, pH, activation status (e.g., CD62P).	Dynamic adjustment of parameters; reject suboptimal batches.
4. Closed-aseptic packaging	Encapsulate PRP in sterile, closed systems (e.g., single-use tubing).	Prevent contamination and ensure traceability.
5. Final QC & storage	Flow cytometry for viability; store at 80 °C or use immediately.	Guarantee potency and safety for clinical use.

### 5.6 Translational Collaboration and Regulatory Innovation

Multidisciplinary collaboration among engineers, clinicians, and regulatory bodies is essential for advancing PRP therapies, with practical relevance enhanced by citing ongoing projects such as those integrating engineers in device design for closed-loop centrifugation systems with AI quality control, alongside clinicians optimizing dosage, as real-world examples of cross-disciplinary work. Establishing international standards for exosome characterization and adopting adaptive trial designs will accelerate clinical validation, while regulatory innovation is crucial to adapt to emerging technologies, ensuring patient access to safe and effective novel therapies. Such translational partnerships, bolstered by tangible collaborations and real-world applications, will bridge the gap from laboratory discovery to clinical application, accelerating the development of next-generation PRP-based treatments.

## ​6. Conclusion​

PRP-based therapies hold immense promise for revolutionizing cardiovascular regenerative medicine by modulating angiogenesis, inflammation, and myocardial repair. However, realizing this vision requires overcoming technical challenges in standardization, safety, and precision delivery. By embracing nanotechnology, synthetic biology, and artificial intelligence, the field can transition from empirical treatments to data-driven, mechanism-specific interventions. Ultimately, PRP’s evolution from a “passive regenerative agent” to an “intelligent precision platform” will redefine the future of cardiovascular care.
